# Inhibition of Stromal PlGF Suppresses the Growth of Prostate Cancer Xenografts

**DOI:** 10.3390/ijms140917958

**Published:** 2013-09-03

**Authors:** Karin Zins, Anita Thomas, Trevor Lucas, Mouldy Sioud, Seyedhossein Aharinejad, Dietmar Abraham

**Affiliations:** 1 Laboratory for Molecular Cellular Biology, Center for Anatomy and Cell Biology, Medical University of Vienna, Vienna 1090, Austria; E-Mails: karin.zins@meduniwien.ac.at (K.Z.); anita.thomas@meduniwien.ac.at (A.T.); trevor.lucas@meduniwien.ac.at (T.L.); seyedhossein.aharinejad@meduniwien.ac.at (S.A.); 2 Department of Immunology, Institute for Cancer Research, The Norwegian Radium Hospital, Montebello, Oslo N-0310, Norway; E-Mail: mosioud@medisin.uio.no

**Keywords:** prostate cancer, placental growth factor, xenograft model, tumor-host interaction, tumor angiogenesis

## Abstract

The growth and vascularization of prostate cancer is dependent on interactions between cancer cells and supporting stromal cells. The primary stromal cell type found in prostate tumors is the carcinoma-associated fibroblast, which produces placental growth factor (PlGF). PlGF is a member of the vascular endothelial growth factor (VEGF) family of angiogenic molecules and PlGF mRNA levels increase after androgen deprivation therapy in prostate cancer. In this study, we show that PlGF has a direct dose-dependent proliferative effect on human PC-3 prostate cancer cells *in vitro* and fibroblast-derived PlGF increases PC-3 proliferation in co-culture. In xenograft tumor models, intratumoral administration of murine PlGF siRNA reduced stromal-derived PlGF expression, reduced tumor burden and decreased the number of Ki-67 positive proliferating cells associated with reduced vascular density. These data show that targeting stromal PlGF expression may represent a therapeutic target for the treatment of prostate cancer.

## 1. Introduction

Prostate cancer is the second most common cancer in males worldwide and the second most common cause of cancer related death among men [1]. Most tumors are primarily treated with surgery or radiation therapy; advanced-stage or widespread disease at initial diagnosis or disease progression after primary treatment is treated with hormone ablation therapy [2]. Although virtually all prostate cancers are initially androgen sensitive and initial therapy can result in significant long-term remission, the development of advanced, malignant, incurable prostate cancer is frequently associated with loss of androgen-dependence and the formation of distant metastases [3]. The term hormone-refractory prostate cancer was traditionally used for tumors that no longer respond to gonadal androgen deprivation therapy [4] and at this stage of disease treatment options are very limited [4].

Prostate tumor growth is accompanied by neovascularization and stimulation of vessel growth from the existing vasculature in a process known as angiogenesis that is required to ensure adequate supply of oxygen and nutrients and removal of waste products from the growing tumor [5]. Angiogenesis is induced by up-regulation of angiogenic growth signals or the corresponding receptors, down-regulation or loss of angiogenic inhibitors or alterations in the intracellular signaling pathways activated by these molecules [6,7]. In prostate tumors, the microenvironment primarily consists of supporting stromal carcinoma-associated fibroblasts (CAFs) that help promote carcinogenesis by remodeling of the extracellular matrix (ECM) and inducing cellular proliferation and angiogenesis [8,9]. Targeting this reactive stroma is currently under investigation as a potential therapeutic modality in prostate cancer therapy [10].

Placental growth factor (PlGF), also known as PGF and endocrine gland-derived vascular endothelial growth factor [11,12] is a member of the vascular endothelial growth factor (VEGF) family of angiogenic molecules [13]. Many cell types produce PlGF, especially when activated or under stress. In addition to the physiological production of PlGF by endothelial cells, vascular smooth muscle cells, inflammatory cells and fibroblasts, several types of tumors express and secrete PlGF [14]. PlGF homodimers bind FLT-1 homodimers (fms-like tyrosine kinase, also known as vascular endothelial growth factor receptor-1 (VEGFR)-1), as well as homodimers of the co-receptors Neuropilin (NRP)-1 and NRP-2 [11,15]. PlGF is also able to transmit signals through FLT-1, without crosstalk with KDR (kinase insert domain-containing receptor, also known as fetal liver kinase (FLK-1) or VEGFR-2) [16]. It is also possible that PlGF heterodimerizes with VEGF [11,16] to transmit angiogenic signals through binding to and activation of the FLT-1/KDR heterodimer receptor complex [16]. PlGF is involved in endothelial stimulation, pathologic angiogenesis and wound healing [17,18] and directly stimulates neovascularization by stimulating the proliferation, migration and survival of endothelial cells [17]. While PlGF is over-expressed in some cancers such as breast and gastric carcinoma, expression is down-regulated in colon and lung carcinoma [19,20]. PlGF is required for the growth of medulloblastoma [21] and also promotes the growth of chronic myeloid leukemia [22]. However, in other tumors PlGF inhibits tumor growth [23]. A screening program to identify angiogenic biomarkers associated with prostate cancer treatment and progression has recently identified increased levels of PlGF to be associated with androgen deprivation therapy [24].

In this study, we identified a role for fibroblast-derived PlGF in the proliferation of human, androgen independent PC-3 prostate cancer cells and investigated the therapeutic potential of siRNA-mediated PlGF blockade *in vivo*.

## 2. Results and Discussion

### 2.1. Results

#### 2.1.1. Murine PlGF Stimulates the Proliferation of PC-3 Cells *in Vitro*

**Figure 1:**
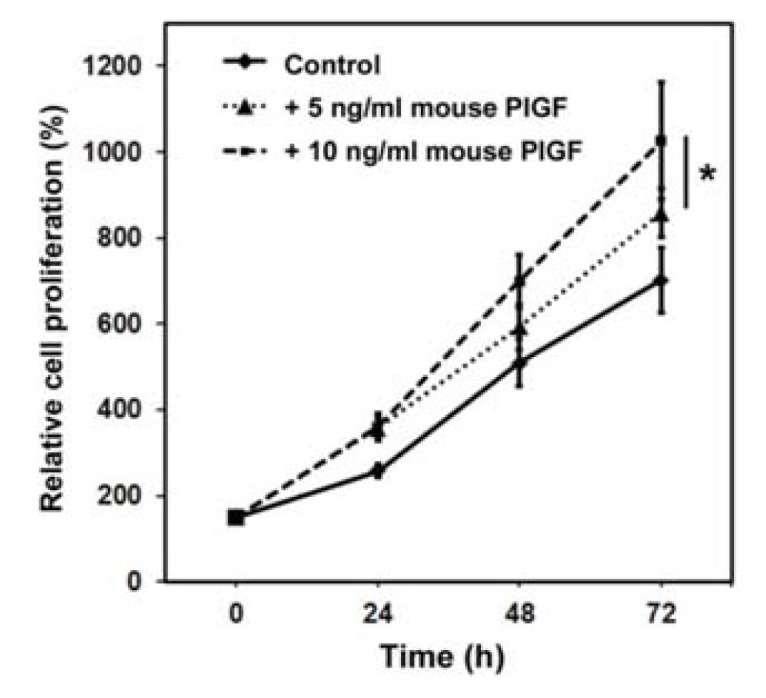
Murine placental growth factor (PlGF) enhances the proliferation of human PC-3 prostate cancer cells. PC-3 cells were incubated with 5 and 10 ng/mL recombinant mouse PlGF. Cell proliferation, measured by WST-1 cell proliferation assays after 24, 48 and 72 h was enhanced in a time-dependent manner. Proliferation was expressed as percentage of corresponding untreated control cells on day 0 (mean ± SD). * cells treated with 5 and 10 ng/mL PlGF were significantly different from control, *p* < 0.05.

Recombinant human PlGF enhances PC-3 prostate cancer cell proliferation (data not shown). To evaluate whether murine PlGF has a direct effect on PC-3 prostate cancer cells and thus constitutes a valid *in vitro* model for the analysis of prostate cancer cell—fibroblast interaction, we cultured cells in 5 ng/mL or 10 ng/mL recombinant murine PlGF and performed a WST-1 cell proliferation assay. Both PlGF treated groups displayed a significantly higher proliferation rate 72 h after treatment compared to untreated controls as shown in Figure 1. No significant difference between groups treated with 5 ng/mL and 10 ng/mL PLGF was observed although proliferation was markedly increased in prostate cancer cells treated with 10 ng/mL compared to 5 ng/mL PlGF. These results show that mouse PlGF enhances PC-3 proliferation in a time-dependent manner, predicting an effect for murine stromal PlGF on PC-3 xenograft growth in mice.

#### 2.1.2. Inhibition of PlGF Expression in Co-Cultured PC-3 and S3T3 Fibroblasts

**Figure 2:**
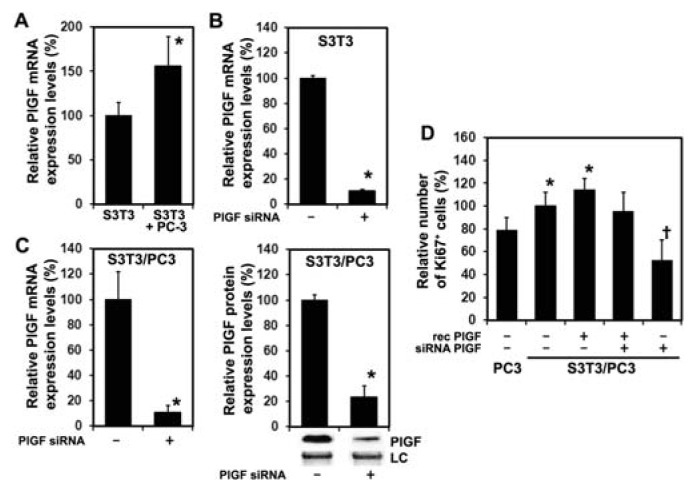
Murine PlGF siRNA reduces PlGF expression of S3T3 fibroblasts. (**A**) Co-culturing S3T3 fibroblasts with prostate cancer cells increases S3T3 PlGF expression. * significantly different from S3T3 alone, *p* < 0.05; (**B**) Transfection with siRNA targeting murine PlGF efficiently reduces endogenous S3T3 fibroblast PlGF expression 24 h following transfection compared to controls transfected with scrambled siRNA. Expression levels are expressed as percentage of corresponding control values (mean ± SD). * significantly different from control, *p* < 0.001; (**C**) In co-cultures of PC-3 with S3T3 fibroblasts transfected with scrambled siRNA, treatment with siRNA targeting murine PlGF reduces murine PlGF expression on the mRNA level 24 h post-transfection as assessed by real time RT-PCR (left panel). * significantly different from control, *p* < 0.001. Quantification of Western blots representatively shown with respective loading control (LC) demonstrates that murine PlGF protein is reduced 24 h post-transfection in co-cultures of PC-3 with S3T3 (right panel). Expression levels are expressed as percentage of corresponding control values (mean ± SD). * significantly different from control, *p* = 0.003. (**D**) Co-culturing S3T3 fibroblasts with prostate cancer cells increases the number of Ki-67 positive PC-3 nuclei when compared PC-3 cells alone. Treatment of PC-3/S3T3 co-cultures with siRNA targeting murine PlGF significantly reduces the number of Ki-67 positive PC-3 nuclei when compared to scrambled controls or PlGF siRNA co-cultures supplemented with 5 ng/mL murine PlGF. Proliferation is expressed as percentage of corresponding control values (mean ± SD). * significantly different from PC-3 cells alone *p* < 0.05; ^†^ significantly different from controls and PlGF supplemented co-cultures, *p* < 0.05.

Human PC-3 cells do not express detectable levels of PlGF as measured by real-time PCR (data not shown). Murine S3T3 fibroblasts express PlGF and expression increases after co-culturing with PC-3 cells (Figure 2A) and PlGF expression can be efficiently reduced by transfection with siRNA targeting murine PlGF (Figure 2B). The role of fibroblast-derived PlGF on the proliferation of PC-3 cells was then examined in co-culture experiments with S3T3 fibroblasts. Transfection with siRNA reduces expression of murine PlGF in co-cultures on the mRNA (Figure 2C left panel) and protein levels (Figure 2C right panel). Co-culturing prostate cancer cells with fibroblasts increased the number of Ki-67 positive PC-3 nuclei and stimulation with 5 ng/mL murine PlGF further increased Ki-67 positive PC-3 nuclei. Reduced S3T3 PlGF expression is associated with significantly decreased numbers of Ki-67 positive PC-3 nuclei that normalizes in the presence of 5 ng/mL murine PlGF (Figure 2D). These data show that S3T3 derived PlGF has a direct proliferative effect on PC-3 cells which can be attenuated in co-cultures by siRNA targeting murine PlGF.

#### 2.1.3. siRNA Mediated Blockade of Host PlGF Attenuates PC-3 Prostate Cancer Growth *in Vivo*

**Figure 3:**
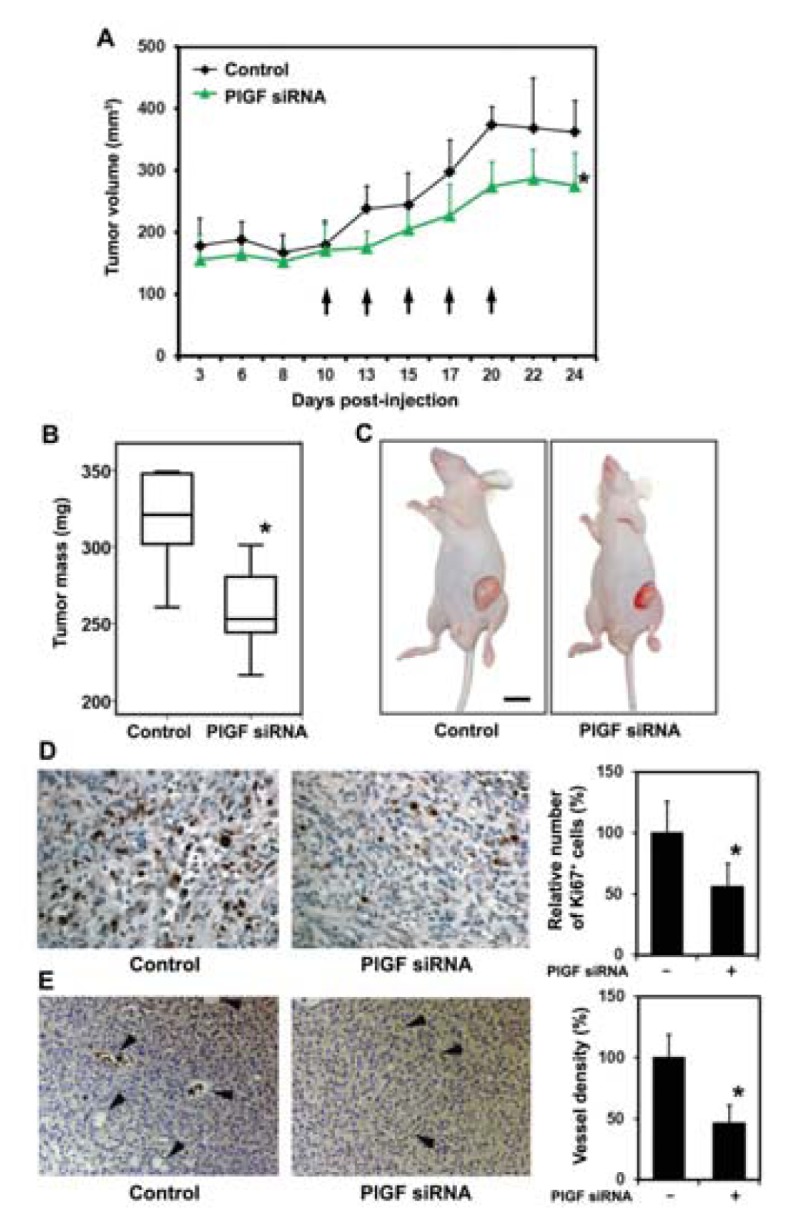
Stromal PlGF blockade reduces growth of prostate cancer xenografts. (**A**) Intratumoral injections of siRNA targeting murine PlGF commencing 10 days following PC-3 engraftment and cycled at the indicated time points (arrows) significantly reduced tumor volumes on day 24. * significantly different from control, *p* = 0.015; (**B**) Tumor mass was significantly reduced in the PlGF siRNA group on day 24. * significantly different from control, *p* = 0.008; (**C**) Representative images of tumor-bearing mice on day 24; (**D**) Representative images show that siRNA targeting murine PlGF reduces tumor proliferation (Ki-67 staining). * significantly different from control, *p* < 0.05; (**E**) Stromal PlGF inhibition reduces microvessel density in the tumors on day 24. Representative images of sections from control tumors and PlGF siRNA treated tumors (arrowheads indicate microvessels; asterisks indicate erythrocytes within vessels) and quantification of microvessel density. Results are expressed as percentage of controls. * significantly different from control, *p* < 0.05.

As shown in Figure 3A, PlGF siRNA treatment markedly suppressed tumor growth compared to the control group treated with scrambled siRNA. Following initiation of therapy on day 10 when tumor volumes were comparable at 171.6 ± 41.3 mm^3^ in the control group and 180 ± 38.6 mm^3^ in the PlGF siRNA group, tumor growth in the control group was higher at all subsequent time points measured at 238.2 ± 37 mm^3^ in the control group compared to 174.71 ± 26.5 mm^3^ in the PlGF siRNA group (day 13), 244.9 ± 51.6 mm^3^*vs.* 205.2 ± 35.2 mm^3^ (day 15), 297.6 ± 51.5 mm^3^*vs.* 227 ± 50.9 mm^3^ (day 17) 374.6 ± 28.4 mm^3^*vs.* 273.8 ± 40.4 mm^3^ (day 20) 368.9 ± 81.1 mm^3^*vs.* 287.3 ± 47 mm^3^ (day 22) and at termination on day 24, 362.4 ± 50.8 mm^3^*vs.* 275.5 ± 52.5 mm^3^.

Tumor weights of the PlGF siRNA treated animals were significantly reduced by 18.6% from 316.9 ± 33.3 mg to 258 ± 29.5 mg when compared to control animals (Figure 3B). Tumor proliferation as assessed by Ki-67 antibody staining shown representatively in Figure 3D was significantly decreased following PlGF siRNA treatment by 44.2% ± 19.1% compared to control tumors. In addition, PlGF siRNA treatment reduced capillary density significantly by 53.9% ± 15.7% capillaries compared to control animals, shown representatively in Figure 3E.

These results show that PlGF siRNA treatment retards the growth of PC-3 xenografts and reduces tumor proliferation and vascular density.

#### 2.1.4. PlGF siRNA Treated Tumors Reduce Expression of Angiogenesis-Related Factors in Host Tissue

**Figure 4:**
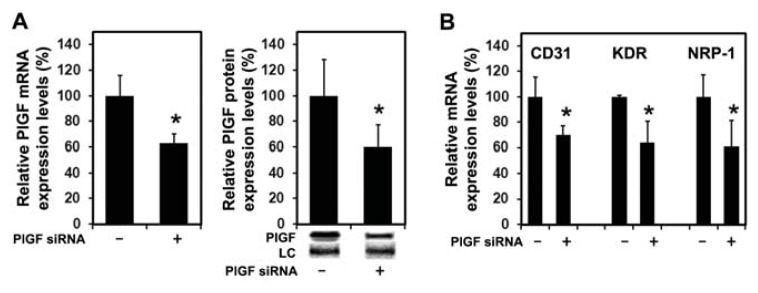
Stromal PlGF blockade reduces angiogenesis-related factors (**A**) Treatment of PC-3 xenografts with siRNA targeting murine PlGF reduces expression of intratumoral murine PlGF mRNA (left panel) and protein (right panel) compared to controls. * significantly different from control, *p* < 0.05; (**B**) Tumoral expression of murine CD31, KDR and NRP1 mRNA was significantly reduced after PlGF siRNA treatment compared to control tumors on day 24. * significantly different from control, *p* < 0.05.

Treatment of PC-3 tumors with PlGF siRNA significantly reduced the expression of stromal derived murine PlGF mRNA (Figure 4A left panel) and protein (Figure 4A right panel). Expression of human PlGF was not detected by real-time RT-PCR in the tumors indicating PlGF expression is not induced in PC-3 cells within the tumor microenvironment. Since the vascular density in PlGF siRNA treated tumors was significantly reduced, we assessed the expression of a panel of molecules related to PlGF signaling and angiogenesis. As shown in Figure 4B, mRNA levels of murine CD31, KDR and NRP-1 were significantly reduced on the mRNA level whereas the expression of VEGF-A and FLT-1 remained unchanged (data not shown).

### 2.2. Discussion

In this study, we identify a direct proliferative effect for PlGF on PC-3 prostate cancer cells and investigated the role of PlGF as a potential target in prostate cancer treatment. Healthy adult prostate cells do not usually express PlGF [25]. The human PC-3 prostate cancer cell line, which is a model for advanced castration-resistant prostate carcinoma, also did not express PlGF mRNA and PlGF protein either *in vitro* or *in vivo*. During prostatic carcinogenesis the stroma undergoes progressive loss of smooth muscle, the primary component surrounding the prostatic ducts of a healthy human prostate, associated with the appearance of carcinoma-associated fibroblasts (CAFs) [8]. CAFs can promote carcinogenesis in initiated but non-tumorigenic human prostatic epithelial cells by stimulating epithelial proliferation, for example [8]. CAFs are thus major constituents of the prostate tumor stroma and both produce PlGF [14] and respond to PlGF through stimulation of FLT-1 [13]. As a result, PlGF mRNA is also expressed in human prostate cancer tissues and PlGF mRNA levels increase after androgen deprivation therapy [24].

Our data from co-cultured fibroblasts and PC-3 prostate cancer cells show upregulation of PlGF expression in fibroblasts suggesting that a cancer cell-derived factor leads to upregulation of PlGF. Fibroblasts are also a source of PlGF in other pathological conditions such as rheumatoid arthritis [26]. In addition to fibroblasts, PlGF expression has also been detected in cultured endothelial cells whereas monocyte/macrophage lineage cells are not known to produce PlGF [27]. Thus, it is possible that endothelial cells in the tumor stroma also contribute to stromal PlGF production in prostate cancer tissue. The importance of stromal PlGF upregulation is also supported by findings in other cancer types. In medulloblastoma cells, a cancer cell-derived factor stimulates production of stromal PlGF associated with a strong tumor dependence on stromal-derived PlGF [21]. This is in line with our findings, showing that blockade of stromal PlGF reduces PC-3 prostate tumor growth. Blockade of stromal PlGF therefore offers the advantage of disrupting a tumor-stroma interaction that contributes to prostate cancer progression. Bypassing the genetically instable tumor cell may also reduce the risk of developing therapy resistance. In this context, identifying the prostate cancer cell derived factor, which increases stromal PlGF expression could be additionally important in disrupting tumor-stroma interaction as has been suggested for medulloblastoma [21]. TGF-β is a carcinoma-produced factor which has the potential to regulate the stromal compartment during prostate cancer progression [28] and is a potential candidate regulator of PlGF expression. Moreover, TGF-β signaling is essential for formation of CAFs in the tumor microenvironment and subsequent tumor promoting effects [29]. Consequently, combined inhibition of PlGF and its cancer cell-derived inducer might offer the maximum benefit in disrupting tumor-stroma interactions. The importance of stromal PlGF is also supported by findings in leukemia, in which loss or inhibition of stromal-derived PlGF prolongs the survival of mice with imatinib-resistant Bcr-Abl1(+) leukemia [22].

PlGF directly stimulates neovascularization by stimulating the proliferation, migration and survival of endothelial cells [17]. Loss of PlGF, although not causing any vascular defects during development, reproduction or normal adult life, impairs angiogenesis in pathological conditions [17]. PlGF^−/−^ fibrosarcomas and embryonic stem cell-derived tumors, for example, are smaller and less vascularized than wild type tumors when implanted in nude mice, underlining the role of PlGF in tumor angiogenesis [17]. PlGF regulates many genes involved in angiogenesis [16], the expression of which could be affected by a PlGF-blockade. PlGF is mitogenic and proangiogenic [30] and blockade of PlGF has resulted in inhibition of angiogenesis and tumor cell motility and inhibited growth and metastasis in some models [19], while in others neutralization of PlGF has had no significant effect on tumor angiogenesis [31]. We observed decreased gene expression of the endothelial cell marker CD31 associated with decreased vascular density in tumors, suggesting that the anti-tumor effect of PlGF blockade is at least partly mediated by PlGF stimulated neovascularization in the PC-3 prostate cancer model. Additionally, we found reduced KDR and NRP1 gene expression levels following PlGF therapy. These changes may also interfere with VEGF-driven angiogenesis, since this could affect the transmission of angiogenic signals. By displacing VEGF from FLT-1, PlGF increases the availability of VEGF which in turn can bind to and activate KDR/VEGFR-2 [16]. Activation of FLT-1 by PlGF can lead to intermolecular transphosphorylation of KDR, thus enhancing KDR-phosphotyrosine levels [16]. It is also possible that PlGF heterodimerizes with VEGF [11,16] to transmit angiogenic signals through binding to and activation of the FLT-1/KDR heterodimer receptor complex [16].

Although PlGF-binding receptors have been extensively studied [19], downstream signaling events and roles in cancer cell survival are not fully understood. A recent study showed that the efficacy of anti-PlGF treatment depends on the presence of functional VEGFR1 (the only known tyrosine kinase receptor for PlGF in tumor cells) but not with antiangiogenesis [32]. In addition, anti-PlGF treatment inhibited growth of a tumor engineered to overexpress the PlGF receptor (VEGFR-1) [31]. Thus, it has been suggested that the role of PlGF in tumorigenesis largely consists of promoting autocrine/paracrine growth of tumor cells expressing a functional VEGFR-1 rather than stimulation of angiogenesis [32]. However, direct inhibition of VEGFR-1 activity did not explain anti-PlGF effects in models pancreatic islet tumors [33] and blockade of VEGFR-1 activity does not affect the rate of spontaneous metastasis formation in different tumor models [34]. Another report showed that genetic ablation of VEGFR-1 signaling in the host did not affect tumor growth [31]. Furthermore, a recent study showed that in medulloblastoma, PlGF signaling via NRP-1 and not VEGFR-1 directly conveys prosurvival signals [21]. These findings suggest that alternative signaling mechanisms for PlGF independent of VEGFR1 exist. Consequently, we cannot rule out that such alternative signaling pathways could account for the PlGF tumor promoting effects in prostate cancer.

Measuring CD31 (also called PECAM-1) revealed decreased mouse mRNA levels in prostate cancer tissue following PlGF blockade. CD31 is highly expressed in the vasculature with approximately one million copies of CD31 on the surface of endothelial cells. CD31 is involved in the initial formation and stabilization of cell-cell contacts at lateral junctions of endothelial cells and transendothelial migration [35]. On the other hand, activated KDR stimulates EC migration survival and growth [36]. The observed reduction of CD31 and KDR mRNA thus provides evidence of less EC and/or less mature vessels within the tumor following PlGF blockade. In addition, mRNA levels of NRP-1, which is not only a co-receptor that enhances the binding of VEGF to KDR but also a PlGF receptor and is thought to play a role in the formation of cell-cell contacts and the permeablilization of blood vessels [37], were also reduced in tumor tissue following PlGF siRNA treatment. From these results, it may be concluded that the reduction of tumor weight by PlGF blockade does involve down-regulation of tumor angiogenesis in prostate cancer.

The role of PlGF in tumor growth and tumor angiogenesis seems to be dependent on the tumor type. In some tumors, PlGF may be important as an angiogenic factor whereas in other tumors it may represent a vital growth factor for tumor survival [21]. Our data support the critical importance of host PlGF in PC-3 prostate cancer and reveals PlGF as a necessary factor for prostate cancer cell growth and in promoting tumor angiogenesis.

Stromal PlGF blockade by siRNA did not lead to complete eradication of PC-3 prostate cancer xenografts. This could be due to low siRNA delivery efficiency and/or the development of unknown mechanisms of resistance after anti-PlGF therapy in prostate cancer. PlGF upregulation has been shown to be a host response to anti-angiogenic therapy [38]. Consequently, a similar mechanism can be proposed for any anti-PlGF therapy. In this study, VEGF-A levels were unchanged following PlGF-blockade. It is possible that a compensatory upregulation of angiogenic factors other than VEGF-A might occur, which could contribute to the observed moderate effect of PlGF blockade on tumor growth in this model. However, since PlGF blockade by an antibody inhibited growth and metastasis of various tumors, including those resistant to VEGF receptor inhibitors, and enhanced the efficacy of chemotherapy [19], PlGF antibody could be used for therapy. Recent phase I, dose-escalation clinical studies of humanized anti-PlGF antibody TB403 also showed that anti-PlGF therapy was well tolerated with minimal side effects [39,40]. Furthermore, anti-PlGF therapy in prostate cancer would likely be used in combination with standard therapies. Nevertheless, recent technological advances in the use of lipid nanoparticles (LNPs) to deliver siRNAs into target cells observed improved anticancer efficacy suggesting that nanomedicine provides novel opportunities to safely deliver genes to treat cancer [41]. In line with this, down-regulation of regulators of the major intracellular recycling pathways of LNP-delivered siRNAs show enhanced cellular retention of LNPs inside late endosomes and lysosomes and increased gene silencing of the target gene. These data suggest that siRNA delivery efficiency might be improved by designing delivery vehicles that can escape recycling pathways [42]. Thus, technologies promoting siRNA delivery to silence PlGF involved in prostate cancer might be beneficial in treatment of this disease, although tumor subtype, stage and the tumor microenvironment certainly will affect the outcome of any PlGF inhibition therapy.

## 3. Experimental Section

### 3.1. Cell Culture and Transfection

Human PC-3 (CRL-1435) and mouse S3T3 fibroblasts (CCL-92; mouse embryonic fibroblasts) were obtained from American Type Culture Collection (ATCC, Manassas, VA, USA) and cultured in Dulbecco’s modified eagle medium (DMEM; PAA, Pasching, Austria) supplemented with 10% fetal calf serum (PAA), 50 U/mL penicillin and 250 μg/mL streptomycin (PAA) in a fully humidified air atmosphere containing 5% CO_2_ at 37 °C. PC-3 cells were co-cultured with S3T3 at a ratio of 1:1 (2.5 × 10^4^ cells per cell line per 6-well plate for RNA isolation and proliferation assays or 2.5 × 10^5^ cells per cell line per 55 cm^2^ plate for protein isolation). Co-cultures were incubated for 24 h in the absence of antibiotics in culture medium and were then transfected with siRNA (50 nM) directed against mouse PlGF (target sequence 5′-GUGUACAUCUUGGAUGAAU-3′) or a scrambled siRNA (sequence 5′-GAAGCAGCACGACTTCTTC-3′) using Lipofectamine reagent (Life Technologies, Grand Island, NY, USA). RNA and protein for real-time RT-PCR or Western blot analysis were isolated 24 h following transfection. All experiments were performed in triplicate.

### 3.2. Cell Proliferation Assays

PC-3 cells were seeded in 96-well plates at a density of 5 × 10^4^ cells/well and starved for 24 h before treatment with 5 ng/mL or 20 ng/mL recombinant mouse PlGF (Sigma-Aldrich, St. Louis, MO, USA). Cell proliferation was determined at 24, 48 and 72 h after treatment using the WST-1 reagent (Roche Diagnostics, Indianapolis, IN, USA) according to the manufacturer’s protocol. Proliferation differences were expressed as percentages of untreated control cells. All measurements were performed in triplicate.

### 3.3. Real Time RT-PCR

Total RNA was isolated from cell cultures in TRIzol reagent (Life Technologies, Grand Island, NY, USA). Tumor samples were snap frozen in liquid nitrogen and then homogenized in TRIzol for RNA isolation. Reverse transcription was performed with MMLV reverse transcriptase (Fermentas, Burlington, ON, Canada) and PCR with FastStart DNA Master SYBR Green mix (Roche Diagnostics, Indianapolis, IN, USA).

The 5′-sense–3′/5′-antisense–3′ primers (VBC Genomics, Vienna, Austria) utilized were mouse β-2 microglobulin CCTCACATTGAAATCCAAATGC/CGGCCATACTGTCATGCTTAAC, mouse PlGF GAGCTTCGGCTTGGGAAGAAG/GTTCCAGAGAGGGGACAAAGG, mouse CD31 CAAAG AAAAGGAGGACAG/GATGACCACTCCAATGAC, mouse KDR GGAGATTGAAAGAAGGAAC/ACTTCCTCTTCCTCCATAC and mouse NRP-1 CCAGAAAACATCCGTCTGGT/CCTTGTTTTCTCGGTGCTTC. LightCycler Software Version 3.5.3 (Roche, Mannheim, Germany, 2001) was used for PCR data analysis. The relative quantification of the signals was done by normalizing the signals of the different genes to β-2 microglobulin as described [43]. Measurements were performed in triplicate.

### 3.4. Western Blotting

Cell lysates were prepared [44] and proteins (50 μg/lane) were separated by 9% SDS-PAGE prior to electrophoretic transfer onto Hybond C super (Amersham Pharmacia Biotech, Buckinghamshire, UK). Blots were probed sequentially with goat anti-mouse PlGF (Santa Cruz, Dallas, TX, USA). Proteins were immunodetected by chemiluminescence (Supersignal-West-Femto, Pierce, Rockford, IL, USA) and quantified by Fusion-CAPT-Software 16.07 (Vilber Lourmat, Marne-la-Vallée, France, 2012). Reversible Ponceau S staining was used as a loading control in Western blotting [45].

### 3.5. Histology and Immunohistochemistry, Immunofluorescence

S3T3 cells were labeled with DilC_18_ (Molecular probes, Life Technologies, Grand Island, NY, USA), co-cultured with PC-3, and cytospins prepared and fixed in acetone at 4 °C for 8 min. Tissue samples were fixed in formalin and paraffin embedded. Sections were rehydrated in graded alcohols and antigen retrieval was performed in a microwave in 0.1 M sodium citrate (pH 6.5). Sections and cytospin slides were blocked in PBS supplemented with 5% horse serum and stained with monoclonal rabbit anti-human Ki-67 (DAKO, Glostrup, Denmark) or a rabbit polyclonal von Willebrand Factor antibody (vWF; Abcam, Cambridge, UK) to evaluate the density of endothelial cells (ECs). Primary antibody was detected by biotinylated secondary antibody (Vector Laboratories, Burlingame, CA, USA) and either Alexa Fluor 488 conjugated streptavidin (Molecular Probes, Life Technologies, Grand Island, NY, USA), then rinsed with PBS and stained with 0.1 μg/mL 4′-6-Diamidino-2-phenylindole (DAPI), mounted in Cityfluor and analyzed on a fluorescence microscope (Zeiss, Thornwood, New York, NY, USA) or horseradish peroxidase-conjugated streptavidin (DAKO), then developed with DAB chromogen (Vector Laboratories, Burlingame, CA, USA). Ki-67 positive proliferating cells and capillaries were counted in 10 consecutive (magnification, ×20) fields per slide and results are expressed as percentages of controls. Co-culture cytospins were analyzed for Ki-67 positive nuclei in DilC_18_ negative cells determined by immunofluorescence.

### 3.6. Xenograft Model

Experiments were approved by the Institutional Animal Care and Use Committee at the Medical University of Vienna. Pathogen-free male athymic nu/nu (nude) mice (Harlan-Winkelmann, Borchen, Germany), 5 weeks of age, were weighed and coded and randomly assigned to two experimental groups (*n* = 6). PC-3 (10^7^) cells in 100 μL Ringer solution were then injected subcutaneously (s.c.) in the left flank. Tumor volumes were calculated as length × width^2^ × 0.5. After 10 days when tumors had developed average volumes of approximately 175 mm^2^, mice were anesthetized (ketamine hydrochloride/xylazine at 55:7.5 mg/kg, s.c.) and 10 μg PlGF siRNA or scrambled siRNA dissolved in 20 μL Lipofectamine reagent (Life Technologies, Grand Island, NY, USA) was injected intratumorally. The treatment was cycled on days 13, 15, 17 and 20. On day 24, the animals were sacrificed and the tumors isolated, weighed and prepared for molecular analyses.

### 3.7. Statistical Analysis

We used the Student’s *t* test and ANOVA to compare the data between the groups. All statistical tests were two-sided. Statistical tests were done with the use of SPSS software (version 20, SPSS Inc., Chicago, IL, USA, 2011). Data are expressed as means ± SD. *p* values <0.05 were considered to indicate statistical significance.

## 4. Conclusions

Our findings indicate that targeting stromal PlGF by siRNAs can cause effective suppression of tumor cell proliferation associated with reduced vessel density and a reduction of tumor weight. Furthermore, this study indicates the importance of therapeutics targeting the tumor stroma by direct inhibition of a growth factor specifically expressed in the tumor microenvironment. Therefore, inhibition of PlGF may provide an attractive target for the design of novel anticancer therapeutics for prostate cancer. Future applications of siRNAs, however, are largely dependent on the development of appropriate delivery vehicles with optimal pharmacokinetics and intracellular stability.
